# Genetic and Mechanistic Evaluation for the Mixed-Field Agglutination in B_3_ Blood Type with IVS3+5G>A ABO Gene Mutation

**DOI:** 10.1371/journal.pone.0037272

**Published:** 2012-05-18

**Authors:** Ding-Ping Chen, Ching-Ping Tseng, Wei-Ting Wang, Chien-Feng Sun

**Affiliations:** 1 Department of Laboratory Medicine, Chang-Gung Memorial Hospital, Taoyuan County, Taiwan; 2 Department of Medical Biotechnology and Laboratory Science, Chang Gung University, Taoyuan County, Taiwan; 3 Molecular Medicine Research Center, Chang Gung University, Taoyuan County, Taiwan; 4 Department of Pathology, School of Medicine, Chang Gung University, Taoyuan County, Taiwan; University of Leuven, Belgium

## Abstract

**Background:**

The ABO blood type B_3_ is the most common B subtype in the Chinese population with a frequency of 1/900. Although IVS3+5G>A (rs55852701) mutation of B gene has been shown to associate with the development of B_3_ blood type, genetic and mechanistic evaluation for the unique mixed-field agglutination phenotype has not yet been completely addressed.

**Methodology/Principal Findings:**

In this study, we analyzed 16 cases of confirmed B_3_ individuals and found that IVS3+5G>A attributes to all cases of B_3_. RT-PCR analyses revealed the presence of at least 7 types of aberrant B_3_ splicing transcripts with most of the transcripts causing early termination and producing non-functional protein during translation. The splicing transcript without exon 3 that was predicted to generate functional B_3_ glycosyltransferase lacking 19 amino acids at the N-terminal segment constituted only 0.9% of the splicing transcripts. Expression of the B_3_ cDNA with exon 3 deletion in the K562 erythroleukemia cells revealed that the B_3_ glycosyltransferase had only 40% of B_1_ activity in converting H antigen to B antigen. Notably, the typical mixed-field agglutination of B_3_-RBCs can be mimicked by adding anti-B antibody to the K562-B_3_ cells.

**Conclusions/Significance:**

This study thereby demonstrates that both aberrant splicing of B transcripts and the reduced B_3_ glycosyltransferase activity contribute to weak B expression and the mixed-field agglutination of B_3_, adding to the complexity for the regulatory mechanisms of ABO gene expression.

## Introduction

ABO is the most important blood group system in transfusion medicine [1]. A specific glycosyltransferase encoded by the *ABO* gene plays an important role in the modification of ABO blood antigen [2], [3]. The ABO locus spans over 18 kilobases (kb) and consists of 7 exons which range in size from 26 to 688 base pairs. Most of the coding sequences lie in exons 6 and 7 [4]. Of the common *ABO* alleles, at least two *A* alleles, one *B* allele and two *O* alleles have been reported [5]. For the *A* alleles, the sequence of *A^1^* is the same as the cDNA clone FY-66-1, while *A^1v^* has a single nucleotide (nt) substitution at position 467 (C to T) that leads to the substitution of proline to leucine. For the *B* allele, the sequences differ from *A* allele in 7 positions at nt 297, 526, 657, 703, 796, 803 and 930. For the alleles *O^1^* and *O^1v^*, they share a single nt deletion (G) at position 261 that results in reading frame shift leading to the appearance of a stop codon at the positions 353–355. However, there are five different nt positions between *O^1^* and *O^1v^* alleles at 297, 646, 681, 771 and 829, respectively.

In addition to the common ABO blood types, distinct blood types with weak expression of A or B antigens on red blood cells (RBCs) have been identified and are designated as A_3_, A_x_, A_el_, B_3_, B_x_, B_el_, cis-AB and B(A), respectively [6], [7]. Most of the *A* and *B* sub-alleles responsible for the formation of subgroups have been identified [8]–[12]. Some of these minor alleles have mutation(s) in the coding sequences of ABO gene and most of the mutations are single nt substitution leading to an amino acid alteration. Defect in RNA splicing also accounts for the occurrence of a number of blood types. In the case of B_3_ with typical mixed-field agglutination of RBCs in the presence of anti-B or anti-AB antibody, a number of genetic alternations have been reported. In Japan, Yamamoto *et al.* reported a B_3_ case with a 1054C>T substitution nears the 3′-end of the *B* allele [13]. Ogasawara *et al.* identified a B_3_ case that is generated through gene conversion mechanism around the nt 646 leading to a 646T>A substitution and lack of B specific polymorphism at nt 657 [14]. In Taiwan, B_3_ individuals have been shown to carry a *B* allele with a G>A mutation at the +5 nucleotide of intron 3 (rs55852701) [15]. Such mutation destroys the consensus of the splice donor site and causes splicing error leading to exon 3 skipping during mRNA splicing. The *B^3^* transcript without exon 3 predicts a B glycosyltransferase that lacks 19 amino acids at the N-terminal segment. The frequency of B_3_ among group AB Chinese persons are approximately 1 in 900 [16]. Although B_3_ is the most common B subtype in the Chinese population, only two articles have been published to address the molecular and genetic basis of B_3_ [15], [17]. The mechanism responsible for the mixed-field agglutination of B_3_ RBCs still awaits to elucidate.

In this study, we comprehensively analyze the splicing transcripts isolated from B_3_ individuals in the Taiwanese population. Notably, we found that the splicing transcripts with only exon 3 deletion are rare. Many of the splicing variants result in reading frame shift and generate non-functional B glycosyltransferase. Through the use of K562 cell study model, we report the first experimental evidence that the IVS3+5G>A mutation alone, independent of the promoter and enhancer activity, is sufficient to produce the typical mixed-field agglutination in the presence of anti-B antibody. The significances of these findings are discussed.

## Results

### IVS3+5G>A is a common B gene mutation in the B3 subtype of Taiwanese population

**Figure 1:**
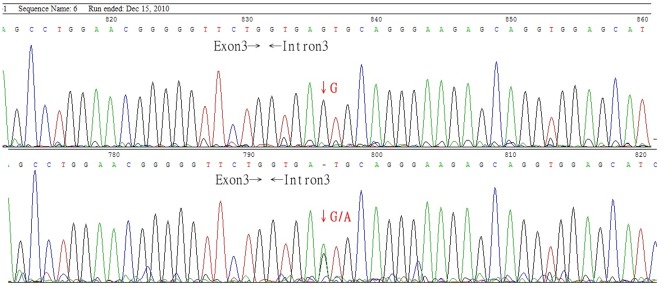
The DNA sequences at the boundary of exon 3-intron 3 in blood type B and B_3_. The DNA sequences between exon 3 and intron 3 in blood type B (upper panel) and B3 (lower panel) case. The B_3_ individuals have been shown to carry a B allele with a G>A mutation at the +5 nucleotide of intron 3.

Previous studies indicate that B_3_ individuals in the Taiwanese population carry the IVS3+5G>A mutation of B allele [15]. To determine whether IVS3+5G>A is common to B_3_, DNA samples from 16 B_3_ individuals were subject to PCR amplification using the primer pairs ABOF303 and B3R120 (Table 1). DNA sequencing revealed that IVS3+5G>A of B allele was present in all of the 16 DNA samples (Fig. 1). Our data therefore confirm previous report by Yu *et al.* and recognize IVS3+5G>A of B allele as a common mutation for all B_3_ cases in the Taiwanese population.

**Table 1:** The primer sequences for generating various ABO alleles cDNA.

Name	Sequence
ABOF303	5′-caaaatgccacgcacttcgacctatgatcc-3′
B3R120	5′-cagagttgagcatgtctacac-3′
cDNA1-F	5′-aaggcggaggccgagaccagacg-3′
cDNA1-R	5′-cctaggcttcagttactcacaac-3′
cDNA2-F	5′-gaattcagccatggccgaggtgttgc-3′
cDNA2-R	5′-tctagaacaacaggacggacaaaggaaacag-3′
ABO exon2-4F	5′-cttggtcttgtttggcatggctgttaggga-3′
ABO exon2-4R	5′-tccctaacagccatgccaaacaagaccaag-3′

### Aberrant splicing transcripts generated by B3 genetic mutation

**Figure 2:**
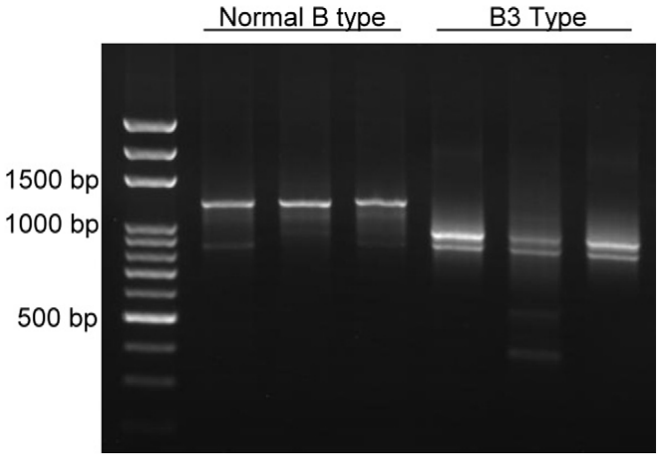
RT-PCR of B1 and B3 transcripts. Buffy coat from the peripheral blood of B1 (n = 3) and B3 (n = 3) cases were subject to total RNA isolation. RT-PCR was then performed to obtain B1 and B3 full-length cDNA using the primer pair ABOF303 and B3R120. The PCR products were analyzed by agarose gel electorphoresis analysis.

According to the theory of RNA splicing, IVS3+5G>A mutation of B allelle is predicted to cause splicing error and generates a B_3_ transcript without exon 3. However, when attempting to isolate full-length B_3_ transcripts from the peripheral blood of B_3_ individuals, at least two major bands with the sizes of 800–900 bp were observed during agarose gel electrophoresis of the RT-PCR products. In contrast, only one major band at approximate 1300 bp was obtained when total RNA from the peripheral blood of B_1_ individual was used (Fig. 2). These data implicate that alternative splicing of B_3_ transcript is likely to occur in a more sophisticated way other than splicing out of exon 3 only.

To unveil the splicing variants in B_3_, we cloned the PCR products from B3 into Zero Blunt II TOPO vector and randomly sequenced 102 independent clones to identify the splicing types (Table 2 and Fig. S1 and S2). Our data revealed that at least 7 types of splicing transcripts were obtained from B_3_ individuals. Exon 2/3/6 deletion represented the most abundant splicing variants with a frequency of 68.6%. In addition, exon 2/3/6 deletion along with intron 6 insertion was the second most abundant splicing variants with a frequency of 11.8%. The frequency of exon 2/3 deletion, exon 2/3/4/5/6 deletion, exon 6 deletion, and exon 2/3/4/6 deletion was 8.8%, 6.7%, 1.0%, and 1.0%, respectively. All these splicing variants resulted in frame shift mutation and produced truncated protein without transferase activity. Notably, only one clone carried the splicing variant with exon 3 deletion that was predicted to generate a truncated but functional transferase (Fig. S2). Our data therefore indicate that aberrant splicing of B allele is common in B_3_ cases and the transcript with exon 3 deletion only was rare with the frequency less than 1%.

**Table 2:** The frequency for the alternative splicing types.

cDNA type	Times of clone
Exon 2, 3, 6 deletion	70
Exon 2, 3, 6 deletion+Intron 6 insertion	12
Exon 2, 3 deletion	9
Exon 2, 3, 4, 5, 6 deletion	7
Exon 6 deletion	1
Exon 2, 3, 4, 6 deletion	1
Exon 3 deletion	1
Total	102

### B_3_ affects surface B antigen expression

Various molecular mechanisms including mutations at the promoter, enhancer and coding sequences have been proposed to associate with phenotypic changes for a number of blood types [18]–[21]. We previously used K562 cells as a cell study model to express *B^1^* cDNA and to investigate ABO antigen expression [22]. Hence, we used the same strategy to stably expressed *B^3^* cDNA under the control of cytomegalovirus promoter and determine the effect of B gene exon 3 deletion on surface B antigen expression.

**Figure 3:**
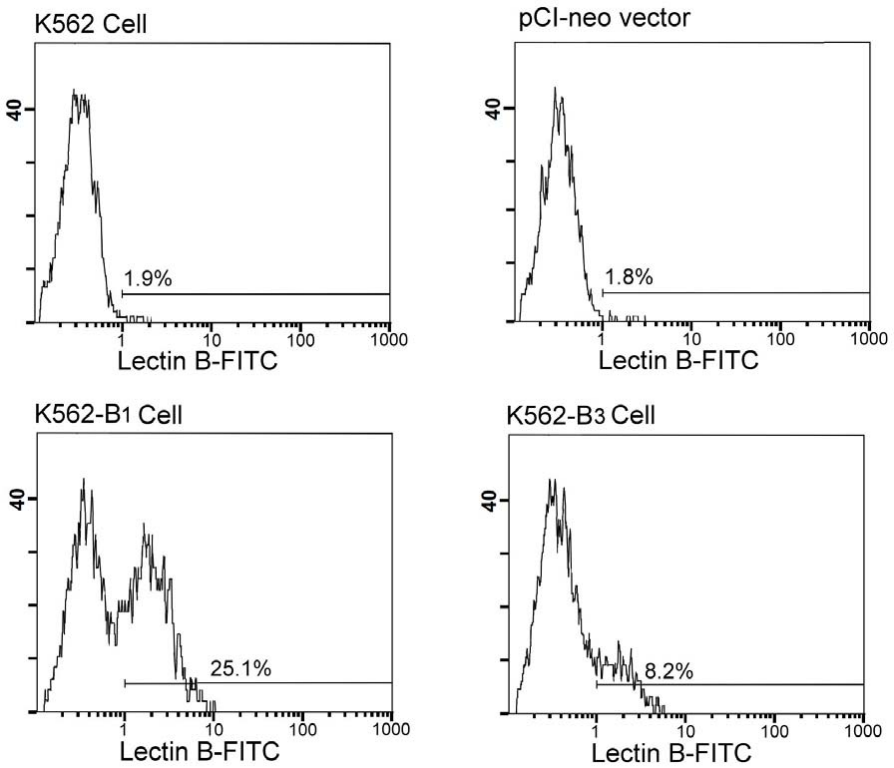
Flow cytometric analysis of B antigen expression. The indicated cell lines were treated with sodium butyrate for 48 h to induce erythroid differentiation. The cells were then incubated with FITC-conjugated BS-I Isolectin B4 from *Bandeiraea simplicifolia*. Flow cytometric analyses were then performed to determine the levels of surface B antigen expression. Representative histograms for the indicated sublines were shown. The FITC-derived fluorescent intensity was displayed on the x-axis on a logarithmic scale and the number of cells on the y-axis.

To compare the levels of antigen expression, the stable lines expressing *B^1^* or *B^3^* cDNA were treated with sodium butyrate to induce erythroid differentiation and surface expression of B antigen was measured by the binding of FITC-conjugated lectin from *Bandeiraea simplicifolia* (BS-I Isolectin B4) followed by flow cytometry analysis. As shown in Fig. 3, the parental K562 cells and the cells transfected with control vector did not express B antigen. In contrast, B antigen expression was increased in the stable cell line expressing *B^1^* or *B^3^* cDNA. The relative percentage of B antigen expressing cells were determined for comparing the levels of B antigen expression in the B_1_ and B_3_ sublines (Table 3). The parental K562 cells that did not express B antigen were used as the negative control whereas the stable cell line expressing *B^1^* cDNA was served as a positive control with the percentage of antigen expressing cells for B_1_ setting as 100%. Accordingly, the relative percentage of B antigen expression for the cells of B_3_ was 40.92% of B1. To further delineate the expression levels of B antigen in the B_1_ and B_3_ sublines, the MFI that represents the total B antigen expression was also compared. We found that the MFI for B_3_ was lower than B_1_ (Table 3). These data thereby demonstrate that exon 3 deletion of B allele causes a decrease in surface B antigen expression.

**Table 3:** The B antigen expression for B and B_3_ subtypes.

Expression constructs	Phenotype	ISBT number	Relative % antigen expressing cellsa	MFIa
K562	O		0	-
B1	B1	B101	100	3431±1530
B3 (intron 3+5G>A)	B3	B303	40.92±11.78*	1831±873*

a The data represent the mean ± S.D. (n = 10) for the indicated transfectants. The mean percentage of B antigen-expressing cells obtained from B101 was used as references and was defined as 100%.

* Student t-test was used for statistically analysis with *p*<0.01 when compared with B101.

### Expression of B3 protein is sufficient to produce mixed-field agglutination phenotype

**Figure 4:**
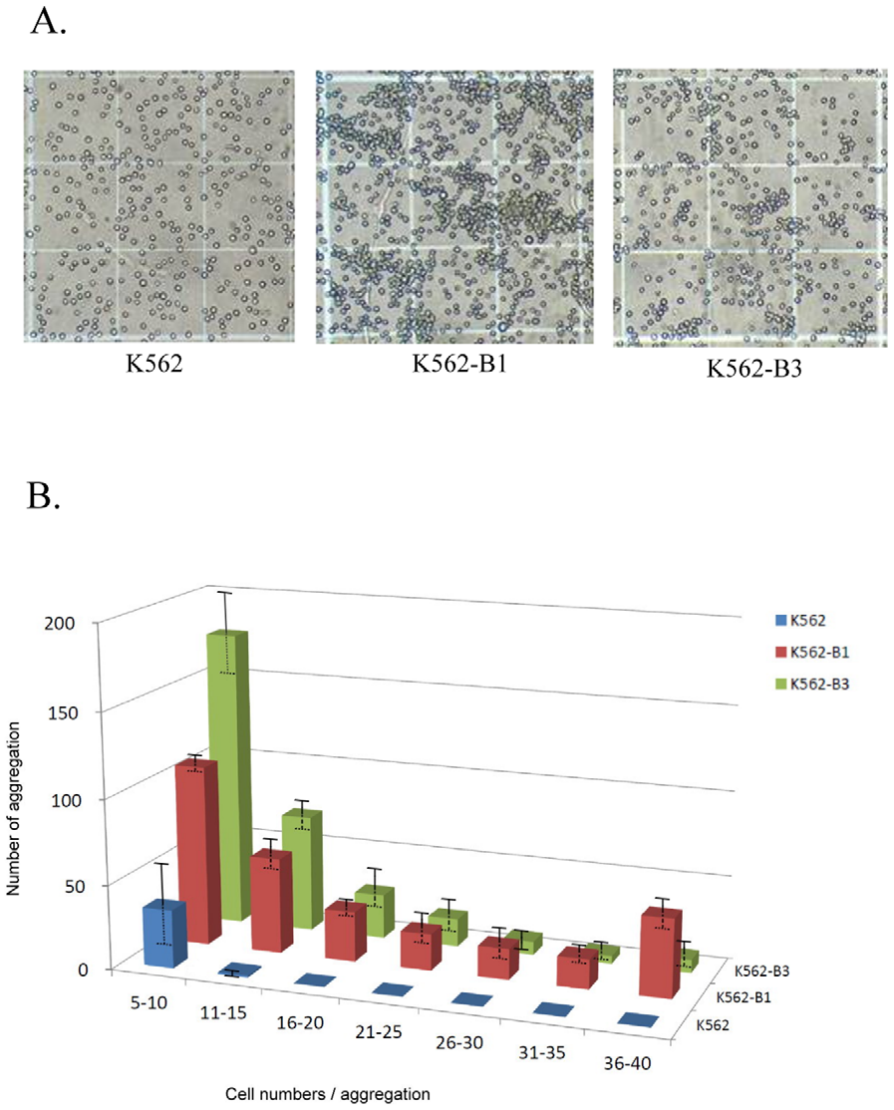
Expression of B3 cDNA is sufficient to induce typical mixed-field agglutination. A. The indicated K562 sublines were treated with sodium butyrate to induce erythroid differentiation. The cells were then incubated with anti-B antibody and cell agglutination was observed using a phase contrast microscope. B. The numbers of aggregation with the indicated number of cells/aggregate were counted. Histogram for the agglutination size distribution was shown. The data represent the mean ± S.D. of three independent experiments. P<0.001 by Kruskal-Wallis test.

Mixed-field agglutination is a typical B_3_ phenotype when reacted with the anti-B antibody. To determine whether expression of exon 3 deletion B_3_ protein alone is sufficient to cause mixed-field agglutination, K562-B_3_ and the control K562 and K562-B1 cells were incubated with the anti-B antibody, respectively (Fig. 4A). No agglutination was observed for the control K562 cells. In contrast, large agglutination appeared when K562-B1 was reacted with the anti-B antibody. Notably, moderate and small size of agglutination that was similar to the characteristic mixed-field agglutination of B_3_ was observed when K562-B_3_ was reacted with the anti-B antibody. Quantitative analysis for the size of agglutination further revealed that K562-B_3_ distributed mainly between 5–10 cells/aggregate (Fig. 4B). Large aggregates with more than 30 cells were only observed for K562-B_1_. These data thereby indicate that expression of exon 3 deletion B_3_ protein is sufficient to produce the mixed-field agglutination phenotype.

## Discussion

B_3_ is a rare blood type that is characterized by mixed-field agglutination of RBCs when reacts with anti-B antibody. In this study, we confirm previous report and identify IVS3+5G>A as the common B_3_ mutation in the Taiwanese population. This mutation causes splicing error and generates mostly non-functional B transcripts. In contrast, the exon 3 deletion transcript that is predicted to generate truncated but functional B transferase accounts for less than 1% of the splicing transcripts in B_3_ individuals. The B_3_ transferase is demonstrated to elicit half of the normal B_1_ activity as revealed by our cell study model analysis. Notably, expression of the exon 3 deletion transcript alone is sufficient to generate mixed-field agglutination in the presence of anti-B antibody. This study thereby provides the first direct evidence for the underlying mechanism of mixed-field agglutination in B_3_ blood type.

Precise pre-mRNA splicing is catalyzed by the spliceosome that recognizes conserved sequences at the exon-intron boundaries and branch point sites [19]. The 5′ splicing site (5′ss) is composed of nine partially conserved nucleotides at the exon-intron boundary, and base pairing to the 5′-terminus of U1 snRNA occurs in this complex [20]. B_3_ was first reported by Yu *et al.* as an intronic mutation at the +5 nucleotide of 5′ splicing site in intron 3. Similar to B_3_, the A_el_ phenotype was found to possess an IVS6+5G>A mutation among Taiwanese and result in weak A [11]. Hence, single nucleotide substitution of the cis-element in pre-mRNA splicing machinery not only associate with the development of various diseases and genetic disorders [23]–[25], but also accounts for the genetic changes associated with ABO blood subtypes.

By analyzing the RNA from B_3_ cases, two different transcripts corresponding to exon 2-exon 7 with skipping of exon 3 (502 bp) and the same structure without the exon 3 and exon 6 regions (367 bp) were previously identified [15]. In this study, we performed RT-PCR and cloned full-length B transcripts from B_3_ individuals to comprehensively analyze the types of splicing transcripts and their relative abundance. Two major findings are noticed during the analysis. At first, the complexity for the splicing transcripts is remarkable and exceeds our expectation. At least 7 different splicing transcripts were identified. Most of these transcripts have exon 3 deletion (101/102), exon 6 deletion (92/102) and exon 3/exon 6 deletion (102/102). Exon 2 deletion that has not been reported previously also contributes a major portion of the splicing transcripts (100/102). The large number of cryptic splicing types occurs in the B_3_ individuals implicates the importance of +5 nucleotide in the 5′ splicing site of intron 3 that is essential for appropriate splicing and generating functional ABO protein.

Secondly, the 7 types of splicing transcripts we identify in this study are present in B3 individuals with different ratio. The exon 2, 3 and 6 deletion (70/102), exon 2, 3, 6 deletion plus intron 6 insertion (12/102), and exon 2 and 3 deletion (9/102) are the three major splicing variants. Due to frameshift and early termination, these three major splicing variants are not predicted to produce functional B glycosyltransferase. In contrast, the transcripts with exon 3 deletion were barely present in B_3_ individual (1/102). Similar to our findings, Huang *et al.* reported a CYP17A1 mutation IVS1+2T>C is associated with 17α-hydroxylase deficiency [26]. When the full-length CYP17A1 minigene containing the intronic mutation was expressed in transfected cells, the majority (>90%) of mRNA transcripts were incorrectly spliced. The IVS1+2T>C mutation therefore abolishes most 17α-hydroxylase/17, 20-lyase enzyme activity by aberrant mRNA splicing to an intronic pseudo-exon, causing a frame shift and early termination. Hence, the rare representation of exon 3 deletion B transcript is likely to associate with the decrease in B glycosyltransferase activity for B_3_ individuals.

**Figure 5:**
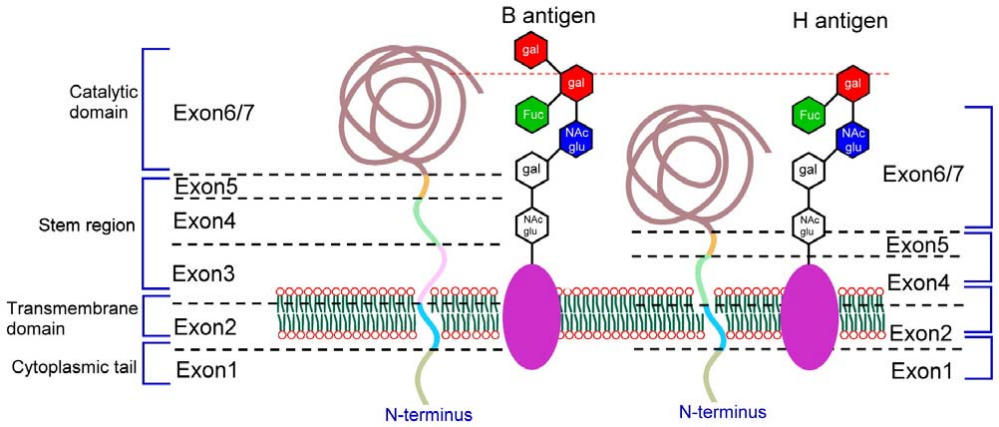
Schematic representation of the B_3_ transferase structure and its interaction with galactose residues. The schematic representation of the B glycosyltransferases is based on previous studies [3], [27]. The relative location of the ABO transferase for the cytoplasmic tail, transmembrane domain, stem region, calalytic domain and the galactose residues are shown. The exons 1 to 7 of the *ABO* gene encoding for the different parts of the B glycosyltransferases are indicated. The B glycosyltransferases catalyze the final step of B antigen synthesis by transferring 1,3-D-galactose residues onto H determinants as the specific acceptor glycoconjugates. As to B_3_, the lack of exon 3 corresponding to the transmembrane and stem region of B glycosyltransferase likely produces B_3_ protein that is unstable and causes a decrease in B_3_ protein expression. It is also likely that, due to the short stem region, B_3_ protein is less flexible and can not efficiently transfer 1,3-D-galactose to the H protein.

At the protein level, the B_3_ transcript with exon 3 deletion is predicted to encode a protein with 336 amino acids. According to the crystal structure of B glycosyltransferase, the protein sequences corresponding to exon 3 are located at the transmembrane and mainly the stem region (Fig. 5). The stem region serves as a flexible tether, allowing the catalytic domain to glycosylate carbohydrate groups of membrane bound H determinants [3], [27], [28]. Hence, the lack of exon 3 likely produces B_3_ protein that is unstable and causes a decrease in B_3_ protein expression. It is also likely that, due to the short stem region, B_3_ protein is less flexible and can not efficiently transfer 1,3-D-galactose to the H protein (Fig. 5). All these effects can result in reduced B antigen expression on the cell surface of RBC. Indeed, when B_3_ cDNA was expressed in the K562 cell study model, B_3_ has only 50% of B_1_ activity in processing surface B antigen expression that provides a molecular basis for the decrease in B antigen expression for B_3_ individuals.

One of the hallmarks of B_3_ is the mixed-field agglutination reaction when B_3_ RBCs are incubated with the anti-B antibody. This phenomenon appears to correlate with the weak B antigen expression on the surface of B_3_ RBCs. Several genetic factors including base insertions, deletions and substitutions mainly in exon 6 and 7 [8], [29], hybrid alleles [30], splice-site mutations [15], [31], variations in enhancer activity [18]–[20], promoter methylation [32], promoter mutations [33] and alternative promoter regions [21] possess the potential to influence A and B glycosyltransferase activity. In this study, we found that mixed-field agglutination can be replicated simply by incubating B_3_-expressing K562 cells with anti-B antibody, indicating that the B_3_ protein alone is sufficient to cause the unique mixed-field agglutination.

Hence, we propose two inter-relative mechanisms that cause a decrease in B surface antigen expression and ultimately mixed-field agglutination phenotype of B_3_. At first, IVS3+5G>A mutation of B gene causes cryptic splicing error and produces at least 7 alternative splicing types. More than 99% of the splicing transcripts do not produce functional protein and less than 1% of the splicing transcripts with exon 3 deletion are predicted to generate functional B_3_ protein. Furthermore, B_3_ protein has only about half of the normal B function. These two interactive mechanisms together account for the weak B antigen expression and ultimately cause the typical mixed-field agglutination of B_3_. This study thereby shed new insight for the genetic and mechanistic effects of IVS3+5G>A on the mixed-field agglutination phenotype associated with B_3_.

Mixed-field phenomenon is usually an indicator of B_3_ blood type when the anti-B or anti-AB antibody was used in the agglutination test. Although it is known that B_3_ individuals in Taiwan carry the *B* allele with a G>A mutation at the +5 nucleotide of intron 3, there is still lack of evidence to demonstrate that B_3_ phenotype is caused by IVS3+5G>A mutation. On the other hand, it is also likely that B_3_ blood type is associated with other genetic changes as reported previously [13]–[17]. The cell study model we reported herein offers a way to confirm whether a particular ABO gene mutation contributes to the indicated blood phenotype. This approach is also applicable in the analysis of other blood types and is thereby important in transfusion medicine.

## Materials and Methods

### Materials

Human leukemic K562 cells with homozygous *O* alleles and surface expression of type H antigens were obtained from American Type Culture Collection (Manassas, VA). The QIAamp RNA Blood Mini Kit was purchased from Qiagen (Valencia, CA). The SuperScript III First-Strand Synthesis System, Zero Blunt TOPO PCR Cloning kit and Lipofectamine 2000 (LF2000) were purchased from Invitrogen (Carlsbad, CA). The pCI-neo mammalian expression vector was purchased from Promega (Madison, USA). The sodium butyrate and FITC-conjugated lectin from *Bandeiraea simplicifolia* (BS-I Isolectin B4) were purchased from Sigma (Saint Louis, MO).

### Collection of blood specimen from B_3_ individuals

Typing of ABO blood type was performed by the standard haemagglutination test. The peripheral blood in K_3_-EDTA tubes was obtained from healthy volunteers as control groups with written informed consent according to the ethical requirements and regulations of the Chang Gung Memorial Hospital Institute Review Board, who approved this study. As to the 16 B3 individuals, the specimens were not individually identified after de-linked process and were analyzed anonymously with the approval of Chang Gung Memorial Hospital Institute Review Board.

### Genomic DNA isolation and PCR

Genomic DNAs of B_3_ individuals were prepared from their peripheral blood cells using the QIAamp DNA Blood Mini Kit (Qiagen GmbH, Hilden, Germany). To determine whether the *B* allele has a G>A mutation at the +5 nucleotide of intron 3, polymerase chain reaction (PCR) was used to amplify the 986 bps DNA fragment of *ABO* gene encompassing the region from exon 2 through intron 3 [15]. Briefly, PCR was set up in a reaction volume of 50 µl containing 1X reaction buffer, 10 nmol of dNTP, 6 pmol of forward and reverse primers (ABOF303 and B3R120, Table 1), 300 ng of genomic DNA, and 1 µl of *Pfu Turbo*® Hotstart DNA Polymerase (Stratagene). The cycling condition was 4 min at 94°C for 1 cycle, 30 sec at 94°C, 30 sec at 58°C, and 45 sec at 72°C for 30 cycles, and 10 min at 72°C for 1 cycle. Subsequently, 5 µl of PCR products were fractionated on the 2% agarose gel and visualized by ethidium bromide staining. The remaining PCR product was used to carry out direct sequencing using the Big Dye Terminator Cycle Sequencing kit (Applied Biosystems, Foster City, CA) and an ABI PRISM 3100 Genetic Analyzer (Applied Biosystems, Foster City, CA) according to the manufacturer's instruction.

### RT-PCR and plasmid construction

Total RNAs were extracted from 1 ml of peripheral blood by QIAamp RNA Blood Mini Kit. Reverse transcription (RT) into complimentary DNA (cDNA) was performed using the SuperScript III First-Strand Synthesis System and the oligo (dT)-15 primers. Briefly, the total RNA (5 µg) was subjected to RT reaction (20 µl) containing 2 µl of 10X RT buffer, 2 µl of 0.1 M dithiothreitol, 1 µl of 10 mM dNTP, 1 µl of 50 µM poly(dT) primer, 1 µl of 40 U/µl RNaseOUT and 1 µl of 200 U/µl SuperScript III reverse transcriptase. The RT condition was 50°C for 50 min and 85°C for 5 min followed by incubation with 1 µl of RNase H for 20 min at 37°C. The RT mixtures were then subject to PCR in a reaction (50 µl) containing 5 µl of RT product, 5 µl of 10X PCR buffer (20 mM MgCl_2_), 4 µl of 2.5 mM dNTP, 2 µl of 10 µM cDNA1-F and cDNA1-R primers (Table 1), and 1 µl of *Pfu Turbo*® Hotstart DNA Polymerase (Stratagene). The cycling condition was 3 min at 95°C for 1 cycle, 30 sec at 94°C, 30 sec at 65°C, and 1 min at 72°C for 35 cycles, and 10 min at 72°C for 1 cycle. The PCR product was cloned into the pCR4Blunt-TOPO vector by the Zero Blunt TOPO PCR Cloning kit. The sequences of the PCR insert were determined using the BigDye® Terminator v3.1 Cycle Sequencing Kit.

### Site-directed mutagenesis

The *B^3^* cDNA was generated by site-directed mutagenesis. Briefly, *B101* cDNA was used as the template to generate two PCR products. At first, a 118 bp DNA fragment of exon 1–4 without exon 3 was amplified by the primer pair cDNA2-F and exon2-4R (Table 1). Then a 975 bp DNA fragment of exon 2–7 skipping exon 3 was obtained by PCR using the primers exon2-4F and cDNA2-R. The HotStart pfu DNA polymerase was used for PCR amplification of cDNA fragments at the cycling condition of 4 min at 94°C for 1 cycle, 30 sec at 94°C, 30 sec at 58°C, and 45 sec at 72°C for 30 cycles, and 10 min at 72°C for 1 cycle. After purification with QIAquick PCR Purification kit (QIAGEN), the 118 bp and 975 bp PCR products were mixed and the second PCR was performed using the primers cDNA2-F and cDNA2-R in the same cycling condition to obtain the *B^3^* cDNA with exon 3 deletion (1134 bp). The 1134 bp PCR product was cloned into the pCRII-TOPO vector by a Zero Blunt TOPO PCR Cloning kit and the sequences were confirmed by DNA sequencing. The inserted plasmid DNA was subsequently digested with *Eco* RI and *Xba* I and inserted into the pCI-neo mammalian expression vector. All constructs were validated by DNA sequencing analysis.

### Stable transfection

The K562 cells (1×10^6^) were routinely cultured in 90% Roswell Park Memorial Institute (RPMI) 1640 medium supplemented with 10% fetal bovine serum, penicillin (50 U/mL) and streptomycin (50 µg/mL), and were transfected with 5 µg of plasmid DNA using LF2000 according to the manufacturer's instructions. The stable clones were selected by antibiotic resistance in the growth medium containing 400 µg/mL of G418 for 2 weeks. The transfectants (3×10^5^) were stimulated by sodium butyrate to induce erythroid differentiation. Forty-eight h later, the cells were harvested and subject to flow cytometry to analyze B and B_3_ antigen expression.

### Flow cytometry analysis

For B antigen detection, K562 cells or the transfectants (3×10^5^) were washed twice with 1X phosphate-buffered saline (PBS) and were incubated for 60 min at room temperature with FITC-conjugated lectin from *Bandeiraea simplicifolia* (BS-I Isolectin B4). After two washes with 1X PBS, the cells were analyzed on a flow cytometer (FACScan). Two variables, the percentage of antigen-expressing cells and the mean fluorescence intensity (MFI), were used for data analysis. MFI is a relative measurment for the amount of antigen expression on the cell surface. The parental K562 cells were used as a reference control.

### Mixed-field agglutination assay

The K562-B_3_ and K562 control cells (3×10^5^) were stimulated by sodium butyrate to induce erythroid differentiation. Forty-eight h later, the cells were adjusted to 5×10^6^/ml and reacted with the anti-B antibody (Immucor Gamma, Norcross, GA) to demonstrate the typical mixed-field agglutination. The number of agglutination in a region of 1 mm×1 mm×0.1 mm was counted manually using haemocytometer under the microscope magnification of 100×.

### Statistical analysis

The data was analyzed using the SPSS 18 software (PASW statistics 18.0). The relative percentage of B antigen expression for the cells of B and B_3_ were compared using the Student t-test. The numbers of aggregate with the indicated number of cells/aggregate were not normally distributed; therefore, the comparison between K562, K562-B and K562-B_3_ was done using nonparametric Kruskal-Wallis one-way analysis of variance. The mean differences with p<0.05 were considered statistically significant.

## Supporting Information

Figure S1 A. The sequences for the cDNA clone skipping of exon 6. Note the direct link for the sequences of exon 5 to exon 7. B. The sequences for the cDNA clone skipping of exons 2 and 3. Note the direct link for the sequences of exon 1 to exon 4. C. The sequences for the cDNA clone skipping of exons 2, 3 and 6. Note the direct link for the sequences of exon 1 to exon 4, and exon 5 to exon 7. D. The sequences for the cDNA clone skipping of exons 2, 3, 4 and 6. Note the direct link for the sequences of exon 1 to exon 5 and exon 5 to exon 7. E. The sequences for the cDNA clone skipping of exons 2 to 6. Note the direct link for the sequences of exon 1 to exon 7. F. The sequences for the cDNA clone skipping of exons 2 and 3, and intron 6 insertion. Note the direct link for the sequences of exon 1 to exon 4 and the intron 6 insertion.
(TIF)

Figure S2 The B3 transcript with exon 3 deletion. The sequences for the cDNA clone skipping of exon 3 were shown.
(TIF)
