# Supplementary figures and images for: Genetic and Mechanistic Evaluation for the Mixed-Field Agglutination in B3 Blood Type with IVS3+5G>A ABO Gene Mutation
Source: PLoS One. 2012 May 18;7(5):e37272. doi: 10.1371/journal.pone.0037272 (PMC3356269; doi:10.1371/journal.pone.0037272)

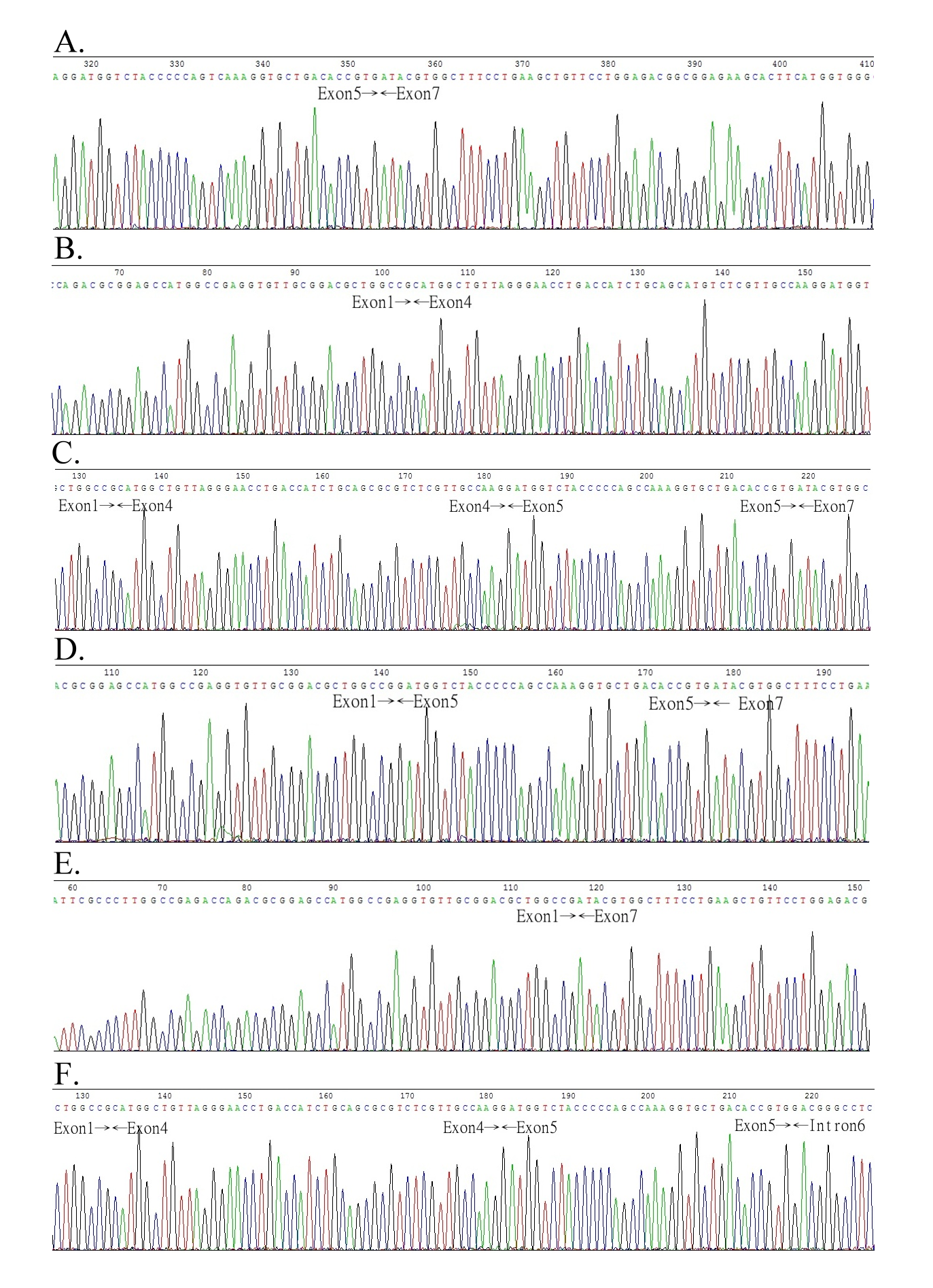

Supplement: Figure S1 — A. The sequences for the cDNA clone skipping of exon 6. Note the direct link for the sequences of exon 5 to exon 7. B. The sequences for the cDNA clone skipping of exons 2 and 3. Note the direct link for the sequences of exon 1 to exon 4. C. The sequences for the cDNA clone skipping of exons 2, 3 and 6. Note the direct link for the sequences of exon 1 to exon 4, and exon 5 to exon 7. D. The sequences for the cDNA clone skipping of exons 2, 3, 4 and 6. Note the direct link for the sequences of exon 1 to exon 5 and exon 5 to exon 7. E. The sequences for the cDNA clone skipping of exons 2 to 6. Note the direct link for the sequences of exon 1 to exon 7. F. The sequences for the cDNA clone skipping of exons 2 and 3, and intron 6 insertion. Note the direct link for the sequences of exon 1 to exon 4 and the intron 6 insertion. (TIF) [file pone.0037272.s001.tif]

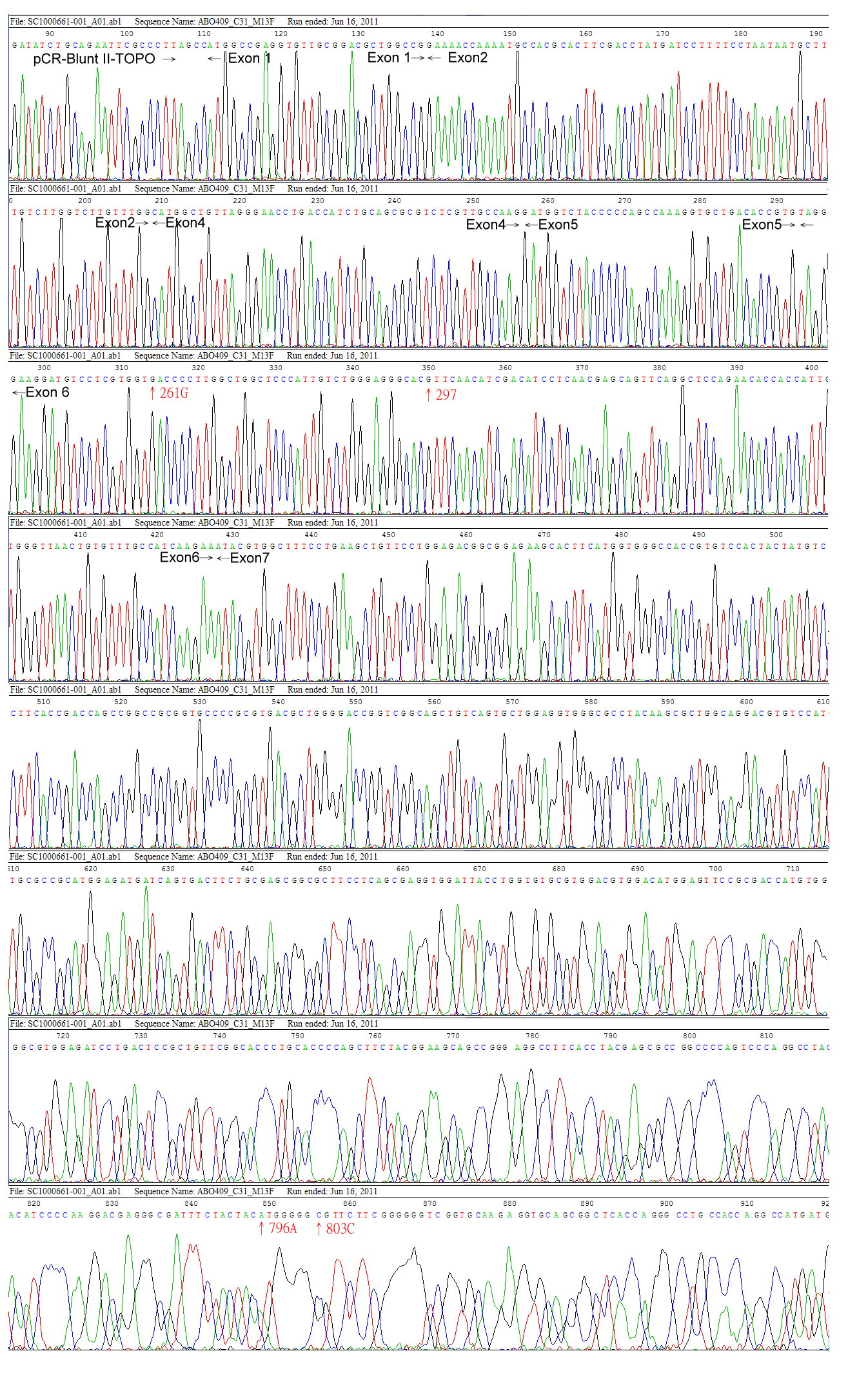

Supplement: Figure S2 — The B3 transcript with exon 3 deletion. The sequences for the cDNA clone skipping of exon 3 were shown. (TIF) [file pone.0037272.s002.tif]
